# Analysis of computed tomography and pathological observations of non-Hodgkin lymphomas with peritoneal, omental and mesenteric involvement

**DOI:** 10.3892/etm.2015.2210

**Published:** 2015-01-23

**Authors:** YUAN-GANG QI, ZE-HUI FANG, YONG HUANG

**Affiliations:** 1 Department of Radiology, Shandong Tumor Hospital, Jinan, Shandong 250117, P.R. China; 2 Department of Radiology, The Affiliated Hospital of Shandong University of Traditional Chinese Medicine, Jinan, Shandong 250012, P.R. China

**Keywords:** abdomen, non-Hodgkin lymphoma, X-ray, computed tomography

## Abstract

The aim of the present study was to evaluate the association between computed tomography (CT) images and the pathological observations of non-Hodgkin lymphoma (NHL) patients with peritoneal, omental and mesenteric involvement. In total, 26 patients suffering from an NHL with peritoneal, omental or mesenteric involvement were reviewed retrospectively, and the observed CT scan characteristics were analyzed. In addition, associations among the CT scan characteristics and the NHL subtypes, including diffuse large B-cell, mantle cell, follicular cell and T-cell lymphoma, were evaluated. The CT scan characteristics of the NHLs with peritoneal, omental and mesenteric involvement included peritoneal cord-like thickening, peritoneal omental nodular and swelling thickening, omental cake-like thickening and mesenteric mass. The probability of peritoneal linear, omental nodular and swelling thickening was found to be higher in diffuse large B-cell lymphoma cases compared with cases of other NHL subtypes (P<0.05). However, the probability of omental cake-like thickening and mesenteric mass was not found to be significantly different among the NHL subtypes (P>0.05). Signs of peritoneal, omental and mesenteric involvement were observed in the CT scans of all the NHL subtypes, particularly in diffuse large B-cell lymphoma cases. Therefore, linear, omental nodular and swelling thickening were characteristic of diffuse large B-cell lymphoma, while omental cake-like thickening and mesenteric mass were observed in all NHL subtypes.

## Introduction

Lymphomas are a group of malignancies that occur in the lymph nodes, extranodal lymphoid tissues and the monocyte-macrophage cell system (1). Lymphomas can be divided into Hodgkin lymphomas and non-Hodgkin lymphoma (NHLs), according to the structure and composition of the tissue pathology. In addition, according to the cell source, NHLs are divided into B-cell and natural killer/T-cell types, while B-cell NHLs can be divided into further subtypes, including diffuse large B-cell, mantle cell and follicular lymphomas (2). In recent years, the incidence of lymphomas has increased gradually and significantly, particularly NHLs (3). The peritoneum, omentum and mesentery are the most complex serosas, and are commonly involved in ovarian, stomach, colon and colorectal cancers, serving as important indicators in prognostic assessment (4). Enlarged lymph nodes are the predominant imaging features of NHL, which indicates that enlarged lymph nodes are the most common imaging manifestation (5), while involvement of the peritoneum, omentum or mesentery is a rare phenomenon. Furthermore, NHLs involving the omentum are not commonly observed in autopsies, but are more common in diffuse large B-cell, mantle cell, follicular cell and T-cell lymphomas (6). Clinically, it is rare that NHL invades the peritoneum, omentum or mesentery, however, lymphoma is considered in the differential diagnosis of extensive peritoneal tumor lesions in computed tomography (CT) images, as the treatment and prognosis differs compared with other types of tumor and the method can improve current understanding of CT signs of the disease. Therefore, the aim of the present study was to investigate the correlation between CT images and pathological observations of NHLs with omental, peritoneal or mesenteric involvement.

## Materials and methods

### General materials

In total, 26 cases of NHLs with peritoneal, omental or mesenteric involvement were admitted to the Shandong Tumor Hospital (Jinan, China) between January 2003 and September 2010. An NHL was pathologically confirmed in all the patients (male, 16; female, 10; age range, 7–70 years; mean age, 46 years). The clinical symptoms included abdominal pain, bloating, an abdominal mass and changes in stool. The study was conducted in accordance with the Declaration of Helsinki and with approval from the Ethics Committee of the Affiliated Hospital of Shandong University of Traditional Chinese Medicine (Jinan, China). Written informed consent was obtained from all the participants.

### Inspection methods

A 64-slice CT scanner (SOMATOM Sensation; Siemens, Erlangen, Germany) was used to scan each patient between the xiphoid process and the pubic symphysis plane, in a conventional supine position (tube voltage, 120 kV; tube current, 240–330 mA; layer thickness, 5 mm; pitch, 5 mm). The patients were administered 500 ml diatrizoate (1%) orally to fill the gastrointestinal tract 30 min prior to scanning. An intravenous bolus injection of the nonionic contrast medium, iohexol (350 mg/ml; dose, 80–100 ml; injection rate, 3 ml/sec), was applied to the elbow for enhanced scanning (Hokuriku Pharmaceutical Co., Ltd., Beijing, China).

### Image analysis

CT images were retrospectively analyzed by two experienced radiologists who were unfamiliar with the pathological situation of the patients. Features observed in the CT scans included cord-like and tumor-like thickening of the peritoneum, abdominal omental nodules, cake-like thickening of the omentum, increased density of the mesenteric fat and a large number of nodules. The CT values of the lymph nodes were significantly more marked following the injection of intravenous contrast medium.

### Statistical analysis

SPSS 11.5 software (SPSS, Inc., Chicago, IL, USA) was used for statistical analysis. Fisher’s exact test for four-fold table was used for the exact test and P<0.05 was considered to indicate a statistically significant difference. The distribution of the NHL subtypes found in the patients was statistically analyzed and the CT scan characteristics of the NHL subtypes were compared.

## Results

### Quantitative distribution of patients with peritoneal, omentalor mesenteric invasion by NHL subtypes

Diffuse large B-cell lymphomas were most commonly diagnosed in NHL patients with peritoneal, omental or mesenteric involvement, while the number of these patients diagnosed with the other three subtypes was significantly lower (Table I).

### Distribution of CT scan characteristics for NHL subtypes

As shown in Table II, 18 patients were diagnosed with diffuse large B-cell lymphoma, of which 15 (83.3%), 13 (86.7%) and 12 patients (66.7%) were found to have cord-like thickening of the peritoneum, omental nodular thickening of the abdomen and abdominal tumor-like thickening, respectively. In addition, of the eight cases with a different NHL subtype, one (12.5%), two (25.0%) and one (12.5%) cases, respectively, were found to exhibit the aforementioned CT scan characteristics. The number of diffuse large B-cell lymphoma cases identified to exhibit the various CT scan characteristics was significantly higher compared with the other NHL subtypes (P<0.05). In total, nine (50.0%) and eight cases (44.4%) with diffuse large B-cell lymphoma were found to exhibit omental cake-like thickening and mesenteric root nodules, respectively, while for the other NHL subtypes, five (62.5%) and six (75.0%) patients exhibited these characteristics, respectively. However, the probability of two or more CT scan characteristics occurring was not found to be statistically significant when comparing the NHL subtypes (P>0.05).

### CT scans of NHL cases with peritoneal, omental and mesenteric involvement

**Figure 1:**
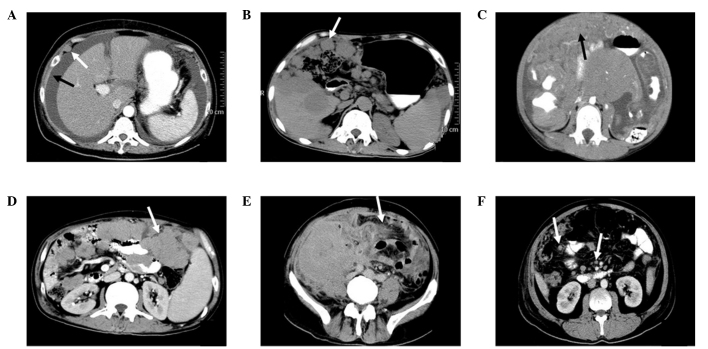
CT scans of non-Hodgkin lymphoma cases with peritoneal, omental or mesenteric involvement. (A) Diffuse large B-cell lymphoma with peritoneal involvement (58-year-old female patient), exhibiting peritoneal nodular thickening (white arrow), cord-like thickening (black arrow) and a significantly thicker peritoneum. Diffuse large B-cell lymphomas with omental involvement, exhibiting (B) peritoneal nodular thickening (white arrow; 48-year-old male patient), (C) omental cake-like thickening (black arrow; 7-year-old male patient) and (D) omental tumor-like thickening (white arrow; 36-year-old male patient). (E) T-cell lymphoma (33-year-old male patient), exhibiting an increased intestinal gap fat density (white arrow). (F) Follicular lymphoma with mesenteric involvement (40-year-old male patient), exhibiting nodules in the mesentery and mesenteric roots (white arrows).

In total, eight cases were found to have peritoneal thickening with significant enhancement, including nodular, cord-like and tumor-like thickening (Fig. 1A). In addition, two cases exhibited omental nodular thickening (Fig. 1B), seven cases showed omental cake-like thickening (Fig. 1C) and one case exhibited tumor-like thickening (Fig. 1D). The fat density of the intestinal interval increased (Fig. 1E) and numerous nodules were observed in the mesenteric root (Fig. 1F).

## Discussion

The peritoneum comprises serosal tissues with a wide scope and complicated structure. Peritoneal primary tumors are less common (peritoneal mesothelioma), while peritoneal secondary tumors are commonly observed in ovarian, stomach, colon, colorectal, pancreatic, endometrial and bladder cancers (7,8). NHLs are a common lymphoid malignancy, identified as the isolation or fusion of lymph nodes through imaging methods. However, NHLs with peritoneal, omental or mesenteric involvement are rare (9), but have been found to exhibit similar CT scan characteristics to peritoneal metastasis. Since NHLs require a completely distinct treatment method from peritoneal carcinomatosis, the correct diagnosis of an NHL with peritoneal, omental or mesenteric involvement is essential.

Lymphomas with peritoneal, omental or mesenteric involvement have been identified to have a lower incidence in females (10–12). In total, 26 patients diagnosed with NHL participated in the present study, including 18 cases (69.2%) of diffuse large B-cell NHL, two cases (7.7%) of mantle cell NHL, two cases (7.7%) of follicular cell NHL and four cases (15.4%) of T-cell NHL. Thus, diffuse large B-cell lymphoma was the most common subtype in NHL patients with peritoneal, omental or mesenteric involvement, which is consistent with the results of a previous study (10).

The omentum consists of a fibrous tissue structure without lymphoid tissues (10); therefore, NHL with omental involvement is rare. At present, the mechanisms underlying lymphoma invasion into omental pathways remain unclear, but have been hypothesized to be similar to gastrointestinal cancer metastasis and the spreading through the transverse mesocolon and stomach mesocolon surface (13). Only 64 cases with peritoneal involvement were identified in the autopsy of 322 NHL cases (6).

CT manifestations of peritoneal lymphoma invasion include diffuse peritoneal cord-like thickening, a smooth surface and significant strengthening, as well as nodular thickening or tumor-like thickening in a number of cases (9,10). CT is the most effective method for the detection of peritoneal thickening, and may even reveal submillimeter nodules. Among the NHL cases, the CT scan results revealed 16 patients with peritoneal cord-like thickening, nine patients with nodular thickening and six patients with tumor-like thickening. All the cases exhibited peritoneal involvement, accompanied by omental thickening or mesangial nodules, with 16 cases also accompanied by a small or moderate number of ascites.

NHLs with omental and mesenteric involvement are commonly accompanied by intestinal lesions, with CT scans revealing the presence of a cake-like soft tissue, nodule or mass (9). In the present study, 18 patients with diffuse large B-cell lymphoma were diagnosed with omentum involvement, including nine cases with omental cake-like thickening, 13 cases with nodular thickening and 12 cases of tumor-like thickening. Peritoneal and omental thickening are not unusual characteristics of NHLs, but are more commonly a feature of peritoneal metastases. The CT scans of the patients were similar, and the diagnostic characteristics were difficult to identify. Changes were identified in the omentum and peritoneum that were reminiscent of tumor samples, which have been rarely reported in patients with metastatic cancer. However, further confirmation is required to clarify whether the observed CT features were due to NHL.

Mesenteric root nodules and increased diffuse mesenteric fat density were also common features of NHLs in the CT scans. The nodules varied in size, had a high occurrence and often oppressed the bowel; however, the nodules were not found in the intestinal canal and obstruction was rarely observed. NHL can invade the mesentry, and their CT signs include obscure gut clearance, increased density and a fixed intestinal canal position. Among the 26 patients, 14 cases were found to have mesenteric root nodules and 12 cases were found to have increased fat density around the bowel. However, mesenteric root nodules and increased diffuse mesenteric fat density are not unique characteristics of NHLs, and are commonly observed in peritoneal carcinomatosis and tuberculosis. Therefore, the CT images obtained showed similar characteristics to these diseases and providing a definitive diagnosis was difficult.

The occurrence of peritoneal cord-like, abdominal omental nodular and abdominal omental tumor-like thickening was higher in diffuse large B-cell lymphoma cases compared with other subtypes (P<0.05). However, the occurrence of omental cake-like thickening and mesenteric root nodules in the NHL subtypes was not found to be statistically significant (P>0.05). Thus, diffuse large B-cell lymphoma is likely to cause peritoneal thickening, nodular thickening of the abdominal omentum and abdominal omental tumor-like thickening. The cake-like omental thickening and mesenteric root nodules between the various NHL subtypes exhibited no characteristic distribution and, due to increased omentum, no significant differences were detected between the mesenteric root nodules between each NHL subtype.

To identify between NHLs with peritoneal, omental and mesenteric involvement and peritoneal metastases, tumor markers can be used. Peritoneal metastases are malignant and exhibit elevated levels of tumor markers. In addition, the peritoneum and omentum exhibit cake-like or nodular thickening, abdominal or retroperitoneal tumors, smaller lymph nodes, limited bowel involvement and obstruction is a common feature. By contrast, the NHL invasion of the peritoneum and omentum may exhibit omental tumor-like thickening, with a wide range of bowel involvement and visible ‘aneurysmal dilatation’ (14), often accompanied by liver or spleen involvement.

Epstein *et al* (16) hypothesized that high-density ascites, along with other CT features, were characteristic of tuberculosis. Intestine, liver and spleen involvement is less common in peritoneal tuberculosis; thus, the conditions may be differentiated and diagnosed by clinical investigation.

CT scans of NHLs with peritoneal, omental and mesenteric involvement revealed peritoneal cord-like, abdominal omental nodular, abdominal omental tumor-like and omental cake-like thickening, as well as increased fat density of the bowel interval and mesenteric gap roots nodules with no specificity. NHLs with peritoneal, omental and mesenteric involvement were more common in diffuse large B-cell lymphoma cases. Therefore, diffuse large B-cell lymphoma is more likely to cause peritoneal cord-like, abdominal omental nodular, abdominal omental tumor-like and omental cake-like thickening, as well as mesenteric root nodules, in various pathological subtypes with no specificity. These CT characteristics are not specific signs of peritoneal and omental invasion by NHL, and other diseases may exhibit the same characteristics. In cases with no typical CT scan characteristics, the CT images of NHLs with peritoneal, omental and mesenteric involvement were difficult to distinguish from CT images of peritoneal metastasis and tuberculosis. In these cases, an aspiration biopsy is required to confirm the diagnosis.

## Figures and Tables

**Table I:** Distribution of non-Hodgkin lymphoma subtypes in patients.

Item	Diffuse large B-cell	Mantle cell	Follicular cell	T-cell
Cases, n	18	2	2	4
Distribution, %	69.2	7.7	7.7	15.4

**Table II:** Comparisons of various computed tomography characteristics observed in patients with different histological subtypes.

Histological subtypes	Peritoneal cord-like thickening	Peritoneal nodular thickening	Peritoneal tumor-like thickening	Omental cake-like thickening	Mesenteric root nodules
Diffuse large					
B-cell, % (n)	83.3 (15/18)	86.7 (13/18)	66.7 (12/18)	50.0 (9/18)	44.4 (8/18)
Other, % (n)	12.5 (1/8)	25.0 (2/8)	12.5 (1/8)	62.5 (5/8)	75.0 (6/8)
P-value	0.001	0.038	0.030	0.683	0.216
